# Repeated thermal stress exposure in *Aedes aegypti* co-infected with *Wolbachia* and dengue virus

**DOI:** 10.1128/msphere.00129-25

**Published:** 2025-09-15

**Authors:** Suk Lan Ser, Fhallon Ware-Gilmore, Nina L. Dennington, Adam Miller, Brianna P. McNulty, Makael L. Harris, Matthew J. Jones, Matthew D. Hall, Carla M. Sgrò, Katriona Shea, Elizabeth A. McGraw

**Affiliations:** 1 Department of Biology, The Pennsylvania State University 118135, University Park, Pennsylvania, USA; 2 The Huck Institutes of the Life Sciences, The Pennsylvania State University 2541, University Park, Pennsylvania, Australia; 3 Department of Entomology, The Pennsylvania State University, University Park, Pennsylvania, USA; 4 School of Biological Sciences, Monash University, Melbourne, Victoria, Australia; University of California Davis, Davis, California, USA

**Keywords:** mosquito, dengue virus, vector-biology, climate change, thermal tolerance, *Wolbachia*, vector control, heat wave

## Abstract

Climate change is increasing the frequency and intensity of heatwaves, affecting the thermal tolerance of mosquitoes and potentially influencing the efficacy of the biological control agent, *Wolbachia*. This study investigates the impact of repeated thermal stress on *Aedes aegypti* mosquitoes co-infected with *Wolbachia* and dengue virus (DENV). We exposed infected mosquitoes (singly and in co-infection) to varying intensities, frequencies, and durations of thermal stress to assess their thermal sensitivity via a “knockdown assay” compared to uninfected controls. Our results demonstrate that co-infection with *Wolbachia* and DENV significantly increases thermal sensitivity, with mosquitoes exhibiting a twofold faster median knockdown time than either singly infected or uninfected controls in most cases. A comparison of mosquitoes with no prior heat exposure to those given a single exposure revealed some evidence of heat hardening, or a slight lengthening of time to knockdown. Additional exposures provided no substantial benefit, however. Extended thermal stress (60 mins) also significantly reduced DENV loads, while *Wolbachia* loads remained stable, indicating that prolonged heat may disrupt viral replication without affecting bacterial symbiosis. These findings suggest that heatwaves could lower vector competence and disproportionately affect DENV-infected mosquitoes in *Wolbachia*-release areas, with implications for biocontrol strategies. Field studies should explore how infection affects mosquitoes’ ability to modulate thermal exposure behaviorally, providing insights for optimizing *Wolbachia*-based control efforts.

**IMPORTANCE:**

Dengue virus (DENV), spread by the mosquito *Aedes aegypti*, is a major global health threat affecting millions of people. This study examines how repeated exposures to heat stress affect the thermal tolerance of mosquitoes infected with DENV and/or *Wolbachia*, a bacterium used for biological control. These repeated exposures mimic the experience of mosquitoes in the wild experiencing heatwaves of increasing frequency under climate change. Our research shows that *Ae. aegypti* co-infected with *Wolbachia* and DENV is more susceptible to thermal stress than singly infected or uninfected mosquitoes. We also demonstrate that multiple independent thermal stress exposures do not exacerbate the effect of infection. Understanding these interactions is essential for predicting how climate change may affect dengue transmission and the resilience of *Wolbachia*-based interventions.

## INTRODUCTION

Global warming is currently increasing average temperatures and is associated with more frequent and intense heatwaves (1). Heatwaves are sudden temperature rises relative to the expected conditions of the area at that time of the year (2). These climate changes can profoundly affect the distribution of vector-borne diseases by altering the habitat suitability of vectors (3, 4). Heatwaves can accelerate mosquito life cycles, shorten the extrinsic incubation period, and increase generational turnover—factors that together can enhance pathogen transmission and potentially lead to more severe disease outbreaks (4–7). Dengue fever is the most prevalent arboviral disease globally, with an estimated 390 million cases occurring yearly (8). The primary vector for dengue virus (DENV) is the mosquito, *Aedes aegypti*, an anthropophilic species highly adapted to urban environments and human settings (9). With rising global temperatures, the geographic range of these mosquitoes is expected to expand, creating a redistribution of the arbovirus transmission (3, 4).

To curb the spread of dengue fever, an innovative vector control strategy involving *Wolbachia pipientis*, an insect endosymbiont, has been recently developed and tested in multiple field release programs globally (10–13). In *Ae. aegypti*, this vertically inherited, self-spreading bacterium limits the mosquitoes’ ability to transmit DENV by interfering with virus replication through a mechanism known as *Wolbachia*-mediated pathogen-blocking (14, 15). The most common use of *Wolbachia* for vector control involves “population replacement” or the release of *Wolbachia-*infected females into populations to allow the bacterium to spread and replace the wild uninfected population with a population at or near fixation with *Wolbachia*. This approach has shown the potential to substantially reduce DENV transmission and, hence, lower the incidence of dengue fever (16). In the most rigorous test of the method conducted in Indonesia, dengue incidence declined by 77% inside the release zones of *Wolbachia* (12). The reduction has ranged from 29.5% to 69% in other localities (10, 11, 17).

Mosquitoes, *Wolbachia*, and DENV all have individual thermal tolerances that may be affected by a changing climate and that may contribute to shifting transmission zones. *Ae. aegypti*, for example, has an operative temperature range between 15.0°C and 35.0°C, with an optimum of 28.0°C (thermal optimum) (18, 19). It is predicted that some regions of the world will become too hot for this mosquito, while others that were previously not habitable, including vast sections of North America, will become ideal. DENV, given that it spends time in mammalian hosts, including those with fever, has an optimal range of 34.0–37.0 ^o^C, with slower replication possible at more extreme temperatures (20). *Wolbachia*’s thermal tolerance is thought to partly depend on its history of adaptation. Its persistence may be influenced by the thermal environment of its host species, as *Wolbachia* must thrive within the physiological limits of its host. For example, *w*Mel and *w*AlbB are the leading strains transfected into *Ae. aegypti* for population replacement (10, 11). The former appears to be less thermally tolerant, possibly given its origins in a cosmopolitan species *Drosophila melanogaster,* with a global temperate range (21, 22). In contrast, the latter strain originated from *Aedes albopictus*, a sister species of *Ae. aegypti,* which, unlike the globally distributed *Ae. aegypti*, is primarily found in tropical and subtropical regions (21, 23).

The effect of infection on the mosquito’s thermal tolerance, however, has been less well studied. Beyond mosquitoes, there is growing evidence that invertebrates, including *Daphnia* and *Drosophila*, are more susceptible to heat stress when they have a microbial infection (24–26). These effects may be direct, due to host immune responses or metabolic costs, or indirect, via microbiota disruption. *Wolbachia* and dengue infect a range of tissues throughout the mosquito’s body, providing ample opportunity to affect local physiology and systemic processes (27, 28). The fitness costs of harboring these microbes under optimal conditions are moderate. In general, DENV can cause some reductions in fecundity and lifespan, although this effect may vary depending on the mosquito’s genetic background, age, and environmental conditions (29, 30). Dengue infection can also sometimes cause behavioral changes by increasing overall activity, altering host-seeking behavior, and increasing probing and blood-feeding attempts (31). Like DENV, *Wolbachia* can also reduce a mosquito’s lifespan, fecundity, and developmental time (27, 32, 33). DENV and *Wolbachia* also trigger active immune responses in the vector (34–36). Recently, our group examined how infection with DENV or *Wolbachia* reduced the mosquito’s ability to respond to thermal stress in a static heat knockdown (KD) assay, leading to more rapid death at a critical maximum temperature (37). The magnitude of this reduction was similar for either single or co-infections of DENV or *Wolbachia*. This led us to hypothesize that there might be a single shared mechanism underpinning the effect, possibly due to indirect effects like energetic tradeoffs or a direct pleiotropic effect given overlapping gene sets in immune and thermal stress responses (34, 35, 38).

While many studies have focused on exposure to higher mean temperatures, less attention has been given to the fact that climate change includes heatwaves and that mosquitoes might experience more than one thermal stress event during their lifetime. In this study, we exposed *Ae. aegypti* to repeated thermal heat stresses of varying frequencies, durations, and intensities to determine the interaction between the cumulative effects of thermal stress and DENV and *Wolbachia* infection. To account for the fact that mosquitoes can fly and seek cooler microclimates during a heatwave, we exposed the mosquitoes to a “short” (10 min) and “long” (60 min) thermal stress event, intended to mimic transient versus extended microhabitat exposures that mosquitoes may encounter in the field. The intensities (i.e., temperatures) of repeated thermal exposure events were 35^o^C (median heat stress) and 40^o^C (upper thermal limit), and were selected because both temperatures are outside the mosquito’s thermal optimum, which would well represent thermal stress (39). While sublethal heat exposures could bring about heat hardening (40–42), given our previous findings, we hypothesized that multiple heat stress events would have a cumulative negative effect on mosquito thermal tolerance in the presence of infection. Our findings can help us understand how DENV infection may affect mosquito survival under changing conditions in the field and whether *Wolbachia*-based biological control programs may cope with heatwaves.

## RESULTS

### Effect of repeated thermal exposure thermal KD

**Fig 1:**
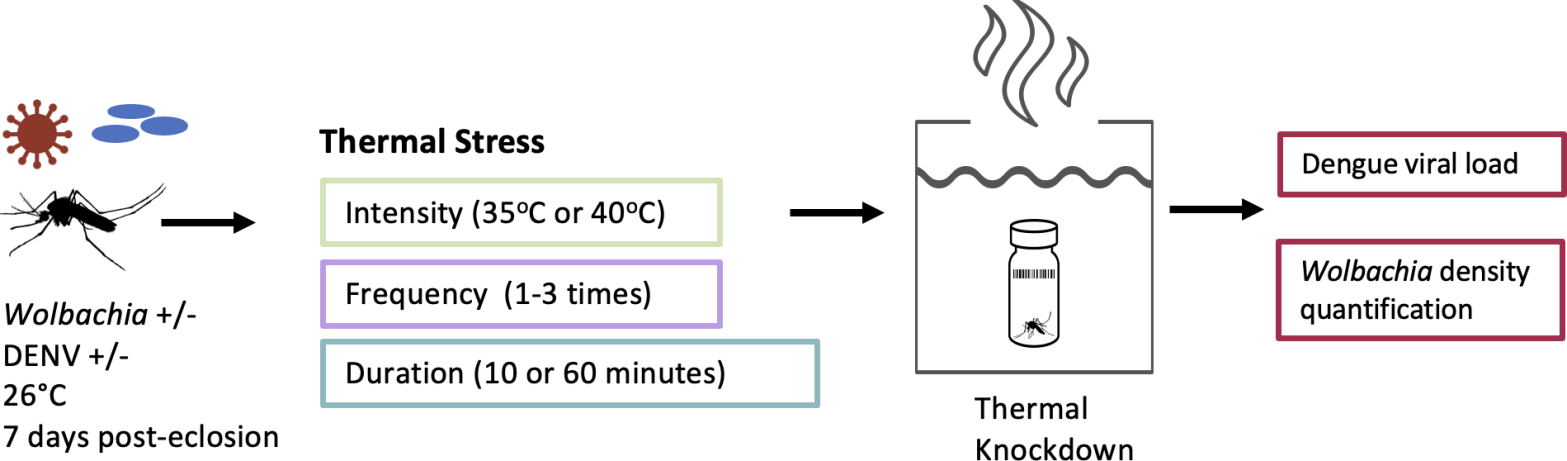
Experimental design of exposing mosquitoes (±DENV, *±Wolbachia*) to thermal stress events with varying intensities, frequencies, and durations, administered at 4-day intervals. At 21 days post-eclosion, mosquitoes underwent a final thermal knockdown assay, followed by collection for dengue viral load and *Wolbachia* density quantification.

We exposed female mosquitoes (±DENV, *±Wolbachia*) to heat shock of different levels of intensity (35°C or 40°C), frequency (one, two, or three heat shocks), and duration (10 or 60 min), as per Fig. 1. In this study, we refer to wild-type mosquitoes as *Ae. aegypti* that are free of *Wolbachia* and not infected with DENV. We tested all treatment groups’ performance in a thermal knockdown (KD) assay at a more extreme temperature, the insect’s CT_max_ (42°C) (37). We aimed to test how differing levels of sublethal thermal exposures would interact with infection to affect subsequent performance. We hypothesized that worsening thermal exposures (increasing frequency, duration, and intensity) would exacerbate previously reported evidence of infection-associated increases in thermal sensitivity (37). The static heat exposure for measuring KD was previously described (37). Briefly, we submerged mosquitoes in glass vials in a 42°C water bath and recorded the time for them to immobilize (KD time) using a barcode scanner. The treatments were blocked over 2 days to manage the scale of the experimental design.

**Fig 2:**
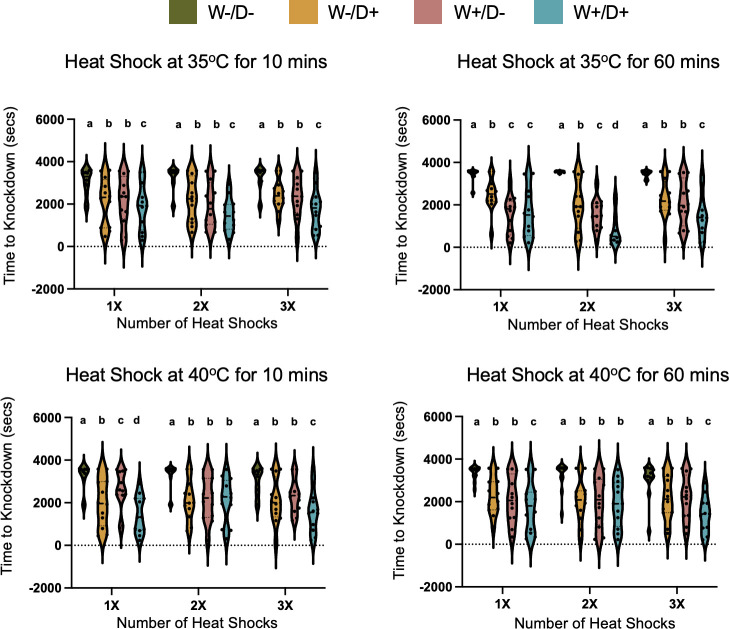
The effect of repeated heat shock events on mosquitoes' thermal sensitivity under varying durations, intensities, and frequencies. The knockdown time is expressed in seconds for mosquitoes (±DENV, *±Wolbachia*), each treatment containing 12 individuals. Violin plots display the distribution of knockdown times, with the thick black bar representing the median and the dotted lines indicating the interquartile range. Individual mosquitoes are represented by dots. Different letters (a,b,c) indicate statistically significant differences (*P* < 0.05) among treatment groups as determined by Tukey’s post hoc test.

We predicted that the effects of multiple heat shocks would compound the negative effects associated with microbial infection. Instead, we found that longer heat shocks (Fig. 2: least-square analysis: “Duration”: *F* = 1.01, df = 1, *P* = 0.31), increasing temperature intensity (“Intensity”: *F* = 0.65, df = 1, *P* = 0.42) and a greater number of exposures (“Frequency”: *F* = 0.49, df = 2, *P* = 0.61) did not influence the thermal sensitivity of the mosquitoes, either wild type or microbe infected. We did show mosquitoes infected with either *Wolbachia* (“*Wolbachia*”: *F* = 128.56, df = 1, *P* < 0.0001) or DENV (“DENV”: *F* = 99.51, df = 1, *P* < 0.0001) exhibited heightened sensitivity to heat across all treatments as per our previous study (37). Additionally, we showed that in 9 out of 12 thermal stress conditions tested, co-infection with both *Wolbachia* and DENV often resulted in an additive effect on thermal sensitivity. Specifically, at 35°C for 10 min, mosquitoes with both infections exhibited median KD time approximately twofold faster than uninfected ones across all exposure frequencies. However, under more extreme heat stress conditions (e.g., 40°C for 10 or 60 min at 2× frequency), co-infected mosquitoes did not differ significantly from singly infected groups, though all infected groups remained more sensitive than uninfected controls. Mosquitoes that were singly infected with either *Wolbachia* or DENV generally had similar average KD times and typically exhibited intermediate thermal sensitivity—greater than uninfected mosquitoes but less than those co-infected with both *Wolbachia* and DENV when exposed to prolonged and intense heat stress (e.g., 40°C for 60 min across all exposure frequencies). However, this pattern was not consistent across all thermal regimes. In 3 out of 12 thermal stress conditions, co-infected mosquitoes did not differ significantly in KD time from singly infected groups. For example, following a single exposure to 35°C for 60 min, mosquitoes co-infected with both *Wolbachia* and DENV did not differ significantly in KD time from singly infected groups. In summary, increasing the exposure to thermal stress by lengthening exposure time and raising the temperature over repeated exposure events did not lead to a change in subsequent thermal performance in the KD assay for wild-type mosquitoes, nor did these exposures interact with microbial infection. We did show that having both *Wolbachia* and DENV infections had an additive effect on KD time—co-infected mosquitoes were more thermally sensitive than singly infected ones when exposed to 40°C for 60 min across all frequencies. However, this additive effect was context-dependent and not consistent across all thermal stress conditions.

**Fig 3:**
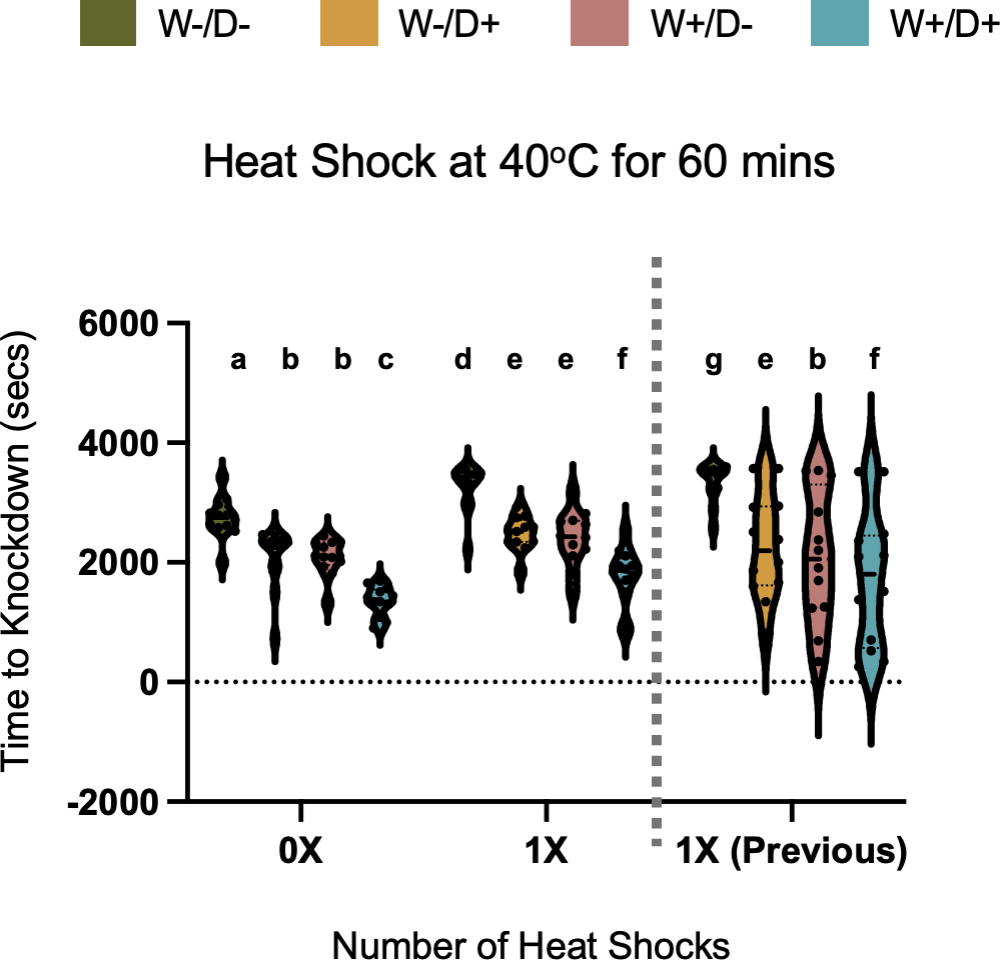
The effect of repeated heat shock events on mosquitoes' thermal sensitivity under two different frequencies (0× and 1×) run in parallel. The knockdown time is expressed in seconds for mosquitoes (±DENV, *±Wolbachia*), each treatment containing 12 individuals. Violin plots display the distribution of knockdown times, with the thick black bar representing the median and the dotted lines indicating the interquartile range. Individual mosquitoes are represented by dots. Different letters (a,b,c) indicate statistically significant differences (*P* < 0.05) among treatment groups as determined by Tukey’s post hoc test. The data beyond the vertical dashed line are from the previous independent experiment (Fig. 2). We include it here to demonstrate that KD time is also greater than 1× although there will likely be some differences in environmental contributions across experiments (mosquito population generation/rearing, virus culture, etc.) that you see in the 1× vs 1×.

To further investigate potential heat-hardening effects, we conducted an additional experiment with a later generation of mosquitoes, comparing individuals exposed to heat shock once to those without exposure at 40°C for 60 min (selected as the most extreme treatment studied). This separate test was added to the experimental design after the fact, to specifically tease apart whether prior heat exposure provided any protective effect against subsequent thermal stress even with a single exposure. We did not include a zero pre-exposure in our original design (Fig. 2) because we expected there to be large cumulative effects from one to three exposures. The effect of a single versus no pre-exposure was significant across all treatment groups (*F* = 26.88, df = 1, *P* < 0.0001), lengthening KD times by ~1.1- to 1.2-fold (Fig. 3). The lengthening of KD time was also present (Fig. 3, beyond dashed line) when we compared each treatment group between 0× in the new experiment and 1× of the previous experiment (data from Fig. 2). We note this with the caveat that this comparison will include additional environmental variation because the mosquitoes were not tested in parallel.

### Effect of repeated thermal exposure on microbe load (DENV and *Wolbachia*)

**Fig 4:**
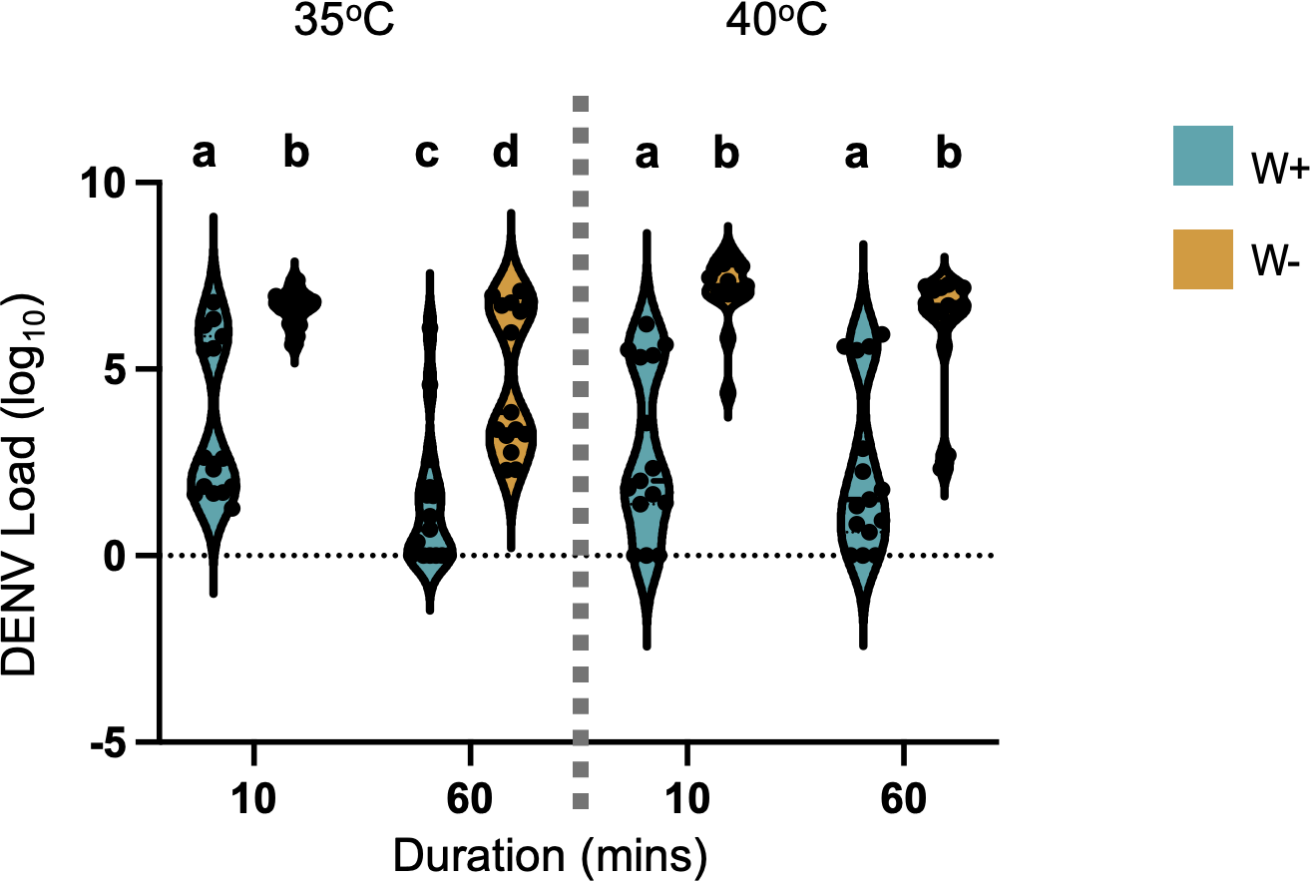
Effect of repeated thermal exposure on DENV viral load in mosquitoes’ head and thorax associated with *Wolbachia* infection. The effect of heat shock events with varying durations (10 or 60 min) and intensities (35°C or 40°C) on DENV-2 viral load (log^10^ copies of DENV per mosquito), with 15 individuals per treatment. These mosquitoes have been exposed to three heat shock events. Violin plots display the distribution of knockdown times, with the thick black bar representing the median and the dotted lines indicating the interquartile range. Dots represent individual heads and thoraxes. Different letters (a,b,c) indicate statistically significant differences (*P* < 0.05) among treatment groups as determined by Tukey’s post hoc test.

We then assessed the effect of repeated heat shock on DENV viral load in mosquitoes (Fig. 4) in association with *Wolbachia* infection. Since the frequency of heat shock events did not significantly alter mosquito thermal sensitivity in most cases, we focused on the most extreme treatment, those exposed to three heat shock events. Mosquitoes exposed to heat shock events with a longer duration (60 min) had a lower viral load compared to those exposed for only 10 min (least square analysis: *F* = 22.59, df = 1, *P* < 0.0001). This effect was significant at 35°C (*F* = 8.76, *P* = 0.0045) but not at 40°C (*F* = 0.91, *P* = 0.35), indicating temperature-specific differences in how prolonged heat exposure influences viral load. The viral load in mosquitoes between those exposed to 35°C or 40°C did not differ significantly (*F* = 0.19, df = 1, *P* = 0.66). *Wolbachia* infection was also shown to reduce DENV viral load by at least an average of twofold in most cases (*F* = 17.24, df = 1, *P* < 0.0001). The same pattern can be observed for the abdomen tissue of the mosquitoes (Fig. S1). In contrast, the *Wolbachia* load was relatively stable, the same across all durations (*F* = 3.00, df = 1, *P* = 0.0857) and intensities (*F* = 1.59, df = 1, *P* = 0.2086) of the heat exposure (Fig. S2). In summary, longer exposures to thermal stress reduced viral loads at 35°C, but *Wolbachia* loads were unaffected.

### Survival

**Fig 5:**
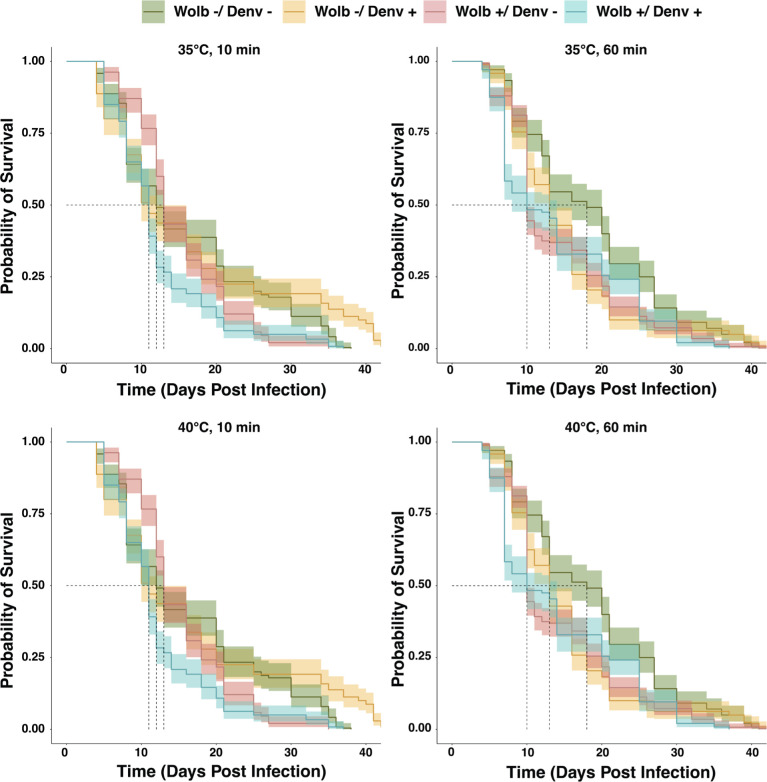
Effect of repeated thermal exposure on the relative fitness of mosquitoes (±DENV, *±wolbachia*). The colored lines represent the probability of survival for 120 individual mosquitoes per treatment group, with the shaded areas representing 95% confidence intervals. The dotted line indicates when 50% of individuals survived.

We then performed a survival assay to analyze the effect of repeated heat shock on female mosquitoes (±DENV, *±Wolbachia*). We assessed their survival daily for 43 days, with data collection beginning on days post-infection (DPI) 0, and presented the results using Kaplan-Meier survival curves stratified by treatment (Fig. 5). Since there were no significant frequency effects, we pooled across these treatments. Additionally, there were multiple replicates within each treatment group, and no significant differences were detected among replicates. We used a pairwise log-rank analysis to compare the survival of these mosquitoes between treatments. The intensity and duration of treatments did not have a consistently significant effect on survival. Survival was, however, affected by infection status. For example, at 35°C and 10 min of prior thermal exposure, mosquitoes that were infected with both DENV and *Wolbachia* died earlier than those that had either one infection or no infection (log-rank, *P* < 0.05) (see Table S1 for information on individual pairwise comparison results). Mosquitoes infected with just DENV had a better survival probability than those infected with just *Wolbachia*. If we looked at the most extreme heat shock conditions, 40°C and 60 min, uninfected mosquitoes had the best survival probability. Double-infected mosquitoes died faster than mosquitoes infected with only *Wolbachia* but did not significantly differ from those infected with only DENV (log-rank, *P* = 0.328). Overall, the survival of these mosquitoes is highly affected by their infection status. Being infected by both *Wolbachia* and DENV almost always lowers the survival probability of mosquitoes relative to those that are either +DENV/*−Wolbachia* or uninfected. In two out of four heat shock treatments that involved longer thermal exposure, the uninfected population had a better survival probability than those single-infected with either *Wolbachia* or DENV.

## DISCUSSION

In this study, we varied the parameters intensity, frequency, and duration to simulate the complexity of thermal exposure events and examine their interactions with infection on mosquito thermal tolerance. Transient exposures to temperatures above the thermal optimum of most organisms, including mosquitoes, can negatively affect their overall physiology, fitness, and behavior (7, 18, 43, 44). At higher temperatures, enzymes and essential proteins involved in regulating various systems such as respiratory, metabolism, nervous, and circulatory become disrupted, causing a cascade of stress responses that is either able to overcome the damage or succumb to it (18, 45, 46). Contrary to our expectations, repeated thermal exposure had no cumulative effect on *Ae. aegypti*’s thermal sensitivity. Being exposed to greater thermal stress by either lengthening the exposure time or increasing the temperature did not make the mosquitoes more susceptible to heat than those exposed to only one thermal event. The absence of cumulative effects may also be attributed to the 4-day recovery period between exposures, which was likely sufficient for physiological recovery. This interval, chosen based on pilot data showing high mortality at shorter intervals, may have allowed mosquitoes to fully recover from each heatwave exposure. It is also possible that the thermal stress conditions in our study were not sufficiently severe to induce significant changes, that is, the mosquitoes are highly robust to thermal stress (47). Heatwaves can persist for days in the field and involve significantly elevated temperatures. While exposures to temperatures higher than 42°C for longer than an hour would have been lethal (37), longer or more frequent exposures at high but sublethal temperatures could be explored in future study designs.

While our initial design focused on repeated exposures, we later incorporated a comparison between mosquitoes with no prior heat exposure and those with at least one pre-exposure to explicitly test the possibility of heat hardening. Heat hardening is a mechanism by which an organism can develop increased thermal tolerance after exposure to a nonlethal, elevated temperature for a certain period. This mechanism has been observed in *Drosophila*, *Apis mellifera,* and other insect species, with each species having its hardening capacity based on their current geographical distribution (41, 42, 48). The test of heat hardening was conducted in a subsequent mosquito generation, but comparable results between the two experiments suggest both consistency of the findings and low involvement of any genetic or environmental differences between generations. We revealed evidence of a moderate hardening effect, with mosquitoes that experienced one prior heat exposure exhibiting increased thermal tolerance compared to those with no prior exposure. The moderate effect was strongest in wild-type mosquitoes, suggesting that prior heat exposure experience provides little protection against future extreme heat events, especially in the presence of infection.

Another key finding from this study was the additive effect of infection on a mosquito’s thermal sensitivity. In all cases, being infected with both DENV and *Wolbachia* made the mosquitoes less tolerant to extreme heat, with a twofold faster median KD time compared to those that were uninfected. In 9 out of 12 cases, mosquitoes infected with just one microbe (either DENV or *Wolbachia*) tended to have a similar average KD time and were more sensitive than uninfected mosquitoes but less sensitive than mosquitoes with both infections. The same pattern was true for the effects on survival. The lower variability observed in uninfected mosquitoes may reflect more stable thermal responses due to unaltered metabolic processes in the absence of bacterial or viral infections. However, it is important to note that this additive effect was not consistent across all thermal stress conditions. These context-dependent outcomes suggest that additive effects of infection may only manifest under certain environmental thresholds or stress intensities. The observed increase in thermal sensitivity in mosquitoes with double infections compared to those with single infections raises several hypotheses for discussion. First, it is essential to note that a previous study by our group did not find any additive effects of *Wolbachia* and DENV infection on mosquitoes’ thermal sensitivity (37). This could be due to two factors: one, the absence of pre-exposure to high temperatures in the original study, which may have prevented the mosquitoes from reaching a threshold where additive effects become apparent. Second, the data from the previous study (37) did trend toward additivity; it may have lacked the statistical power to confirm it. The current study shows clear additive effects in most but not all treatment combinations, suggesting that a single pre-exposure may push the mosquitoes into a condition that reveals these interactions more strongly. These data also show that *Wolbachia* does not confer protection against thermal stress induced by DENV, by reducing virus load through the action of viral blocking. One possible explanation of this additive effect relates to energetic trade-offs. The presence of *Wolbachia* and DENV may significantly burden the mosquito’s immune system and resources, given its propensity to react to both microbes (34, 49–51). This dual burden could become overwhelming when combined with thermal stress, as activating these immune pathways could interfere with the mosquito’s ability to cope with additional stressors. *Wolbachia*, as an obligate intracellular bacteria, lacks specific metabolic pathways, and its consumption/use of the host’s resources, such as amino acids and cholesterol, could deplete essential resources needed for managing thermal stress (52, 53). This competition for resources might be amplified under heat stress conditions, leading to the additive effects we observe.

Another possible hypothesis for an additive effect involves direct pleiotropic effects, where genes and pathways involved in coping with microbial infections might overlap with those required for thermal stress management. For example, infection with *Wolbachia* and DENV activates critical components of the mosquito’s immune response, including RNA interference (RNAi) pathway, the Toll pathway, the Imd (Immune deficiency) pathway, and the JAK/STAT pathway (34, 49–51). These same immune pathways can also be triggered by thermal stress (54–56). Heat shock proteins (HSPs), such as Hsp70 and Hsp90, are involved in managing thermal stress and responding to various other stressors, including anoxia, crowding, and dehydration (57–59). It is possible that these HSPs and other generalized stress proteins like reactive oxygen species may be produced to help manage microbial infections in mosquitoes (35, 60). When these genes are activated or suppressed to address the infections, their effects on thermal stress management can be counterproductive. For instance, upregulating responses to *Wolbachia* or DENV might enhance pathogen defense but could simultaneously interfere with the mosquito’s ability to regulate heat stress, thus explaining the additive effects seen in our study. Conversely, downregulating these responses to improve thermal tolerance might compromise the mosquito’s ability to combat infections. While our previous study hypothesized these mechanisms (37), one novel finding in this study is the manifestation of additive effects. This suggests that pre-exposure to thermal stress may reduce the mosquito’s physiological capacity to balance multiple stressors. Among the mechanisms discussed, resource depletion and competition between immune and stress-response pathways appear most consistent with observed additivity. Future studies should aim to quantify the extent of resource consumption by both microbes under varying thermal conditions to confirm this hypothesis.

Interestingly, while thermal stress exposure did not affect *Wolbachia* load in mosquitoes subjected to multiple thermal stress events, we found that extended exposure (60 min) significantly reduced viral loads compared to shorter exposure (10 min). This phenotypic reduction in viral load could reflect a decrease in viral replication, an increase in mosquito antiviral activity, or a combination of both. DENV has an optimal temperature range for replication. Deviations from this range, high or low, can disrupt the replication cycle, leading to decreased viral loads (20, 61). Additionally, DENV replication relies on temperature-sensitive viral enzymes, such as RNA-dependent RNA polymerase (RdRp). High temperatures can affect the activity and stability of these enzymes, further contributing to reduced replication rates (62). Therefore, prolonged heatwaves in the field could potentially reduce vector competence by lowering the viral load in mosquitoes exposed to extended periods of thermal stress. This suggests that rising temperatures may inadvertently enhance the effectiveness of *Wolbachia*-based biocontrol strategies by selectively reducing DENV transmission in mosquitoes exposed to prolonged heat stress. If this pattern holds under field conditions, it could have important implications for vector control programs, particularly in regions experiencing more frequent and intense heatwaves due to climate change.

Our study suggests *Ae. aegypti* exhibit a degree of thermal tolerance, likely due to slight heat hardening or inherent robustness, which may help them withstand the effects of heatwaves. However, the magnitude of this effect appears limited under our experimental conditions. It also suggests that mosquitoes infected with *Wolbachia* and DENV may be especially susceptible to thermal stress, which is consistent with findings in other host-pathogen systems where co-infection or immune activation increases susceptibility to environmental stressors (63, 64). Given the rarity of mosquitoes infected with DENV during an outbreak (1–2%) (65), the thermal sensitivity could potentially limit the survival of these individuals with DENV in *Wolbachia* release zones, enhancing *Wolbachia’s* efficacy without significantly affecting *Wolbachia* persistence. Mosquitoes infected with DENV, but without *Wolbachia,* may also be limited by their thermal tolerance, a welcome result under rising temperatures. In the field, these effects on host fitness—singly and additively—will play their part in shifting transmission zones and *Wolbachia* efficacy just as much as the individual thermal tolerance ranges of the mosquito, *Wolbachia*, and virus. Future studies are needed to test the effects of infection and co-infection in the field, where mosquitoes can regulate temperature exposure by behavioral modification. These findings underscore *Wolbachia*’s potential role in climate-adaptive vector control strategies, highlighting the need for integrated approaches that account for environmental variability in disease management.

## MATERIALS AND METHODS

### Mosquitoes

*Ae. aegypti* mosquitoes infected with *w*AlbB strain of *Wolbachia* were obtained from Zhiyong Xi (Michigan State). In 2017, the *w*AlbB strain was backcrossed into AFM, the wild-type *Wolbachia*-free line of *Ae. aegypti* obtained from Mérida, Mexico. As this work was carried out about 3 years later, there could be some small genetic differences between lines contributing to phenotypes. The wild-derived line served as a negative control for all experiments. Both lines were reared under standard conditions: 26°C, 60% relative humidity, and a 12-h light/dark photoperiod. During the larval phase, larvae were fed fish food (TetraMin) *ad libitum*. As adults, mosquitoes had access to 10% sucrose.

### Virus cultivation and mosquito blood-feeding

All experiments were performed using DENV-2 strain ET-300 (GenBank accession number EF440433.1) grown in *Ae. albopictus* C6/36 cells (Sigma), as previously described (66).Briefly, C6/36 cells were cultured in RPMI 1640 medium (Life Technologies) supplemented with 10% heat-inactivated fetal bovine serum, 20 mM HEPES buffer (Sigma-Aldrich), and 1% penicillin-streptomycin (Life Technologies). The cells grown to 80% confluence in T75 flasks were then inoculated with DENV-2. Flasks were incubated at 27°C for 7 days, and the supernatant was harvested. The supernatant was mixed in a 1:1 ratio with human blood just prior to mosquito feeding.

Adult female mosquitoes, 7 days post-eclosion, were sugar-deprived for 24 h prior to feeding. The mosquitoes were fed using double-chamber glass feeders covered with pig intestine sausage casing that were warmed to 37°C using a water bath. The final virus concentration in the blood was 1.6e^7^ DENV copies/mL for the thermal KD experiment comparing no prior heat exposure to one heat exposure, and 3.25e^7^ DENV copies/mL for the multiple heat exposure thermal KD experiment. All DENV-negative mosquitoes were fed a 1:1 ratio of blood (without virus) and RPMI 1640 cell culture media to serve as a negative control. Mosquitoes were anesthetized on ice post-feeding, and only those that were fully engorged were retained for subsequent experiments. Fed mosquitoes were sorted into 32 oz paper soup cups with mesh lids and provided cotton balls soaked in 10% sucrose that were changed daily. Environmental rearing conditions were as above for the stock lines.

### Thermal disturbance

All female mosquitoes were then exposed to heat stress with varying intensities (35°C or 40°C), frequencies (one, two, or three heat shocks), and durations (10 or 60 min). With the variables “DENV infection status,” “*Wolbachia* infection status,” “Intensity,” “Frequency,” and “Duration,” there was a total of 48 treatment groups (Fig. 1). The first exposure to heat stress was carried out at 4 DPI, and subsequent heat shocks then occurred every 4 days (DPI 4, 8, and 12). A 4-day interval between exposures was chosen to ensure physiological recovery while enabling the evaluation of cumulative stress responses over multiple exposures. Pilot studies with 2- or 3-day intervals exhibited substantial mortality. Our repeated exposure design also means that when comparing mosquitoes after a single heat shock versus three, for example, they also differ in age. To specifically test the potential for heat hardening, an additional thermal KD experiment was later incorporated using a subsequent mosquito generation (three generations after the initial thermal KD experiment), reared under the same laboratory conditions mentioned above. This experiment followed the same thermal disturbance protocol, comparing mosquitoes with no prior heat exposure (0×) to those exposed to a single heat stress event (40°C, 60 min). Both 0× and 1× mosquitoes were tested for KD time at the same adult age to ensure age-matched comparisons.

### Thermal KD assay

After the above variable exposures to treatments, we then examined the mosquito’s response to thermal stress using a static heat shock assay as described in references 37, 37. Thermal KD experiments were conducted at 42°C (a temperature representing mosquitoes' critical thermal maximum) as determined empirically in a previous study (37) for 60 min. The assay was carried out over 2 days post-blood feed (2 days after the final thermal disturbance) to manage the scale of the design. Mosquitoes from each treatment were randomly selected and placed in individual 40 mL glass vials with solid plastic lids. The vials containing each mosquito were then attached to a vertical plastic board in groups of 48 using anchored clips. The boards were then immersed in a water bath heated to 42°C, and mosquitoes were given a 60-s acclimation period. Mosquitoes were monitored visually for immobility, and time to thermal KD was scored using Brady labels and a TriColor Scanner (Worth Data Inc., Santa Cruz, CA, USA). The immobility of these mosquitoes was confirmed by tapping on the glass vials and visual inspection. These mosquitoes were then collected, and tissues were dissected for DENV and *Wolbachia* quantification (below).

### Survival assay

After blood-feeding, the mosquitoes that were sorted and kept under standard conditions were assessed daily for their survival until all mosquitoes were dead (43 days), with the data collection beginning on DPI 0. These mosquitoes were also subjected to the thermal disturbance regime as above (Fig. 1). Each treatment group (a total of 48) had 60 individuals divided into 4 cups of 15 subjected to heat shock events with the same regime described above. After exposing them to heat stress, these mosquitoes were returned to chambers set at 26°C, their base temperature.

### Mosquito nucleic acid extraction

Individual whole mosquitoes from the thermal KD assays were dissected for their various tissues (salivary gland, midgut, and carcass) that were placed in 300 µL of TRIzol reagent (Sigma-Aldrich). Samples were homogenized on a Bead Ruptor Elite (Omni International, USA) using a 2.8-mm ceramic bead. Total RNA was extracted with the Direct-zol RNA 96 Magbead Zymo Kit (Zymo Research) according to the manufacturer’s protocol. RNA was eluted in 50 µL RNase-free water and then treated with 5 units of DNase I (Sigma-Aldrich) at room temperature for 15 min, followed by inactivation with 50 mM EDTA at 70°C for 10 min. To measure Wolbachia DNA, RNA, and DNA extractions were performed using the column-based Direct-zol DNA/RNA miniprep kit. RNA was eluted into 50 µL RNase-free water, followed by DNA elution in 50 µL Direct-zol DNA elution buffer.

### DENV quantification

DENV was quantified using TaqMan Fast Virus 1-step Master Mix (Thermo Fisher Scientific) in 10 µL reaction volumes with DENV-2 specific primers and probes for qRT-PCR as per previous (67). The following protocol was used: reverse transcription at 50°C for 5 min, followed by 95°C for 20 s, and amplification cycling at 95°C for 3 s and 60°C for 30 s. A standard reference curve of known concentration of DENV-2 genomic fragment was used for absolute qRT-PCR ( Table S2). The DENV-2 genomic fragment was inserted into a plasmid and transformed into *Escherichia coli* as described (67). The linearized and purified fragment was serially diluted, ranging from 10^6^ to 10^2^ copies, and was used to create a standard curve of DNA amplification. The standard curve was run on duplicates on each 96-well plate, along with negative controls.

### *Wolbachia* quantification

*Wolbachia* (*w*AlbB) quantifications by qPCR were performed on a LightCycler 480 Real-Time PCR system (Roche) using previously published primers and probes specific to *w*AlbB and the mosquito ribosomal subunit 17 housekeeping gene (*RpS17*) (68). The sequences of the primers and probes used in this study are listed in Table S2.

DNA samples were amplified using PerfeCTa Multiplex ToughMix (Quanta, 95147-250) following the manufacturer’s protocol. Each qPCR reaction was prepared in a total volume of 20 µL, containing 5 µL of PerfeCTa Multiplex ToughMix, forward and reverse primers, and probes for both wAlbB and RpS17, along with 2 µL of DNA template. Cycling was performed using the LightCycler 480 Real-Time machine, with one cycle at 95°C for 30 s, followed by 40 amplification cycles of 95°C for 3 s, 60°C for 30 s, and a final melting curve analysis. Target gene to housekeeping gene ratios were calculated using the 2^−ΔCt^ method (69).

### Statistical analysis

All statistical analyses were performed using R (version 4.3.2) or JMP Pro (version 18.1.0, SAS Institute Inc., Cary, NC, 1989–2025), depending on the assay. For the thermal KD assay, analyses were conducted in JMP Pro. KD time was analyzed using mixed-effect models, with least squares estimation applied for model fitting via maximum likelihood. KD time was treated as the response variable, while *Wolbachia* infection, DENV infection, frequency, intensity, and duration of thermal stress were included as fixed effects. Statistical comparisons among treatment groups were performed using Tukey’s post hoc tests to assess interactions. The same statistical approach was applied to analyze *Wolbachia* and DENV loads, with either DENV load or *Wolbachia* load as the response variable. For the survival assay, Kaplan-Meier survival curves were generated in R, stratified by treatment groups to visualize survival differences. Pairwise comparisons were conducted using the log-rank test to determine significant differences between treatment groups.

## Data Availability

The data that support the findings of this study are available in Figshare at https://doi.org/10.6084/m9.figshare.27175671.

## SUPPLEMENTAL MATERIAL

Supplemental Material msphere.00129-25-s0001.docx Supplemental figures and tables.

ASM does not own the copyrights to Supplemental Material that may be linked to, or accessed through, an article. The authors have granted ASM a non-exclusive, world-wide license to publish the Supplemental Material files. Please contact the corresponding author directly for reuse.
