# Supplementary material for: Repeated thermal stress exposure in Aedes aegypti co-infected with Wolbachia and dengue virus
Source: mSphere. 2025 Sep 15;10(10):e00129-25. doi: 10.1128/msphere.00129-25 (PMC12570470; doi:10.1128/msphere.00129-25)
Supplement: Supplemental Material — Supplemental figures and tables. [file msphere.00129-25-s0001.docx]

**Supplemental Figures**


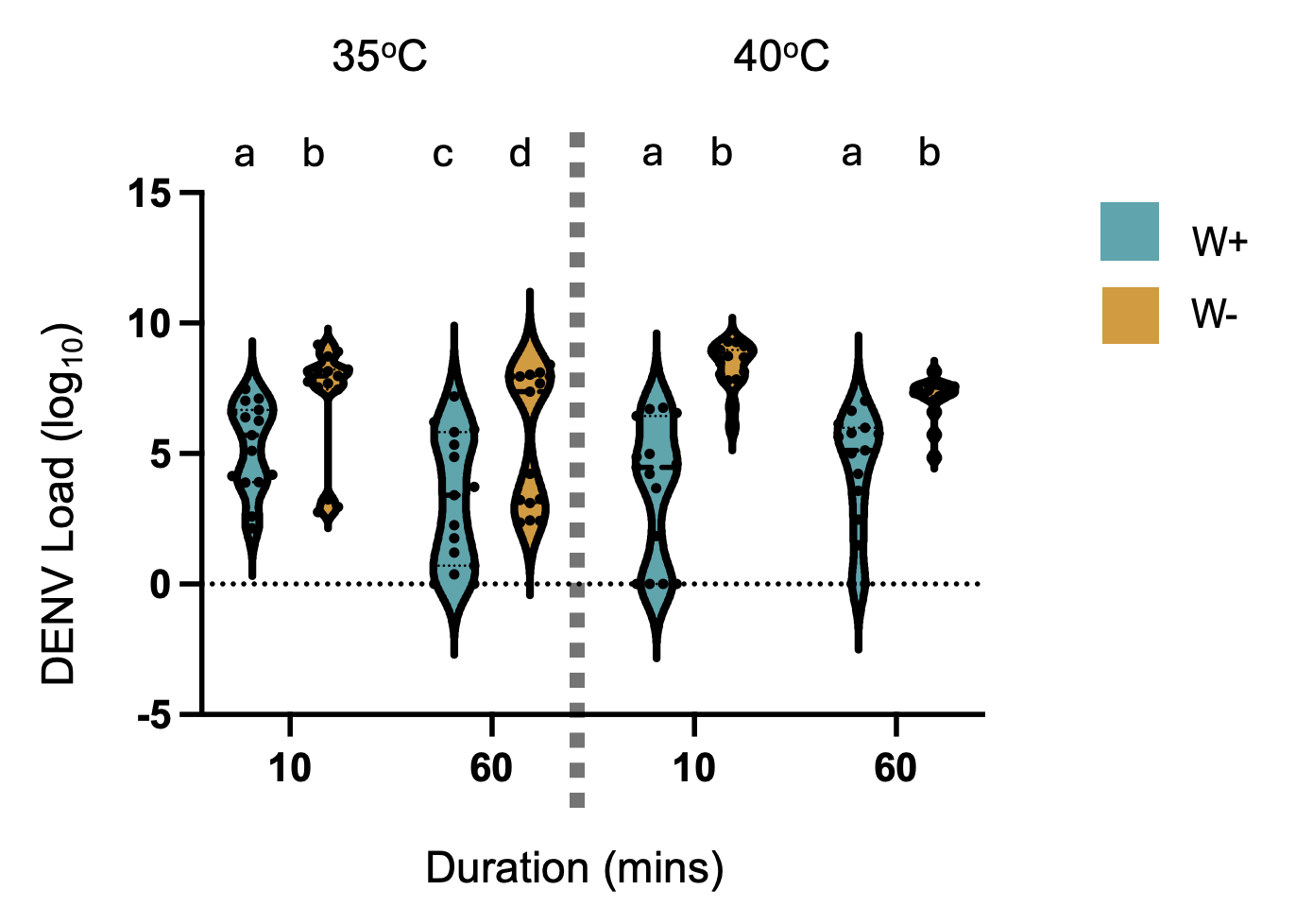


**Figure S1.** Effect of repeated thermal exposure on DENV viral load in mosquitoes’ abdomens in association with *Wolbachia* infection. The effect of heat shock events with varying durations (10 or 60 minutes) and intensities (35°C or 40°C) on DENV-2 viral load (log^10^ copies of dengue virus per mosquito), with 15 individuals per treatment. These mosquitoes have been exposed to three heat shock events. Violin plots display the distribution of knockdown times, with the thick black bar representing the median and the dotted lines indicating the interquartile range. Dots represent individual abdomens. Different letters (a,b,c) indicate statistically significant differences (p<0.05) among treatment groups as determined by Tukey’s post hoc test.


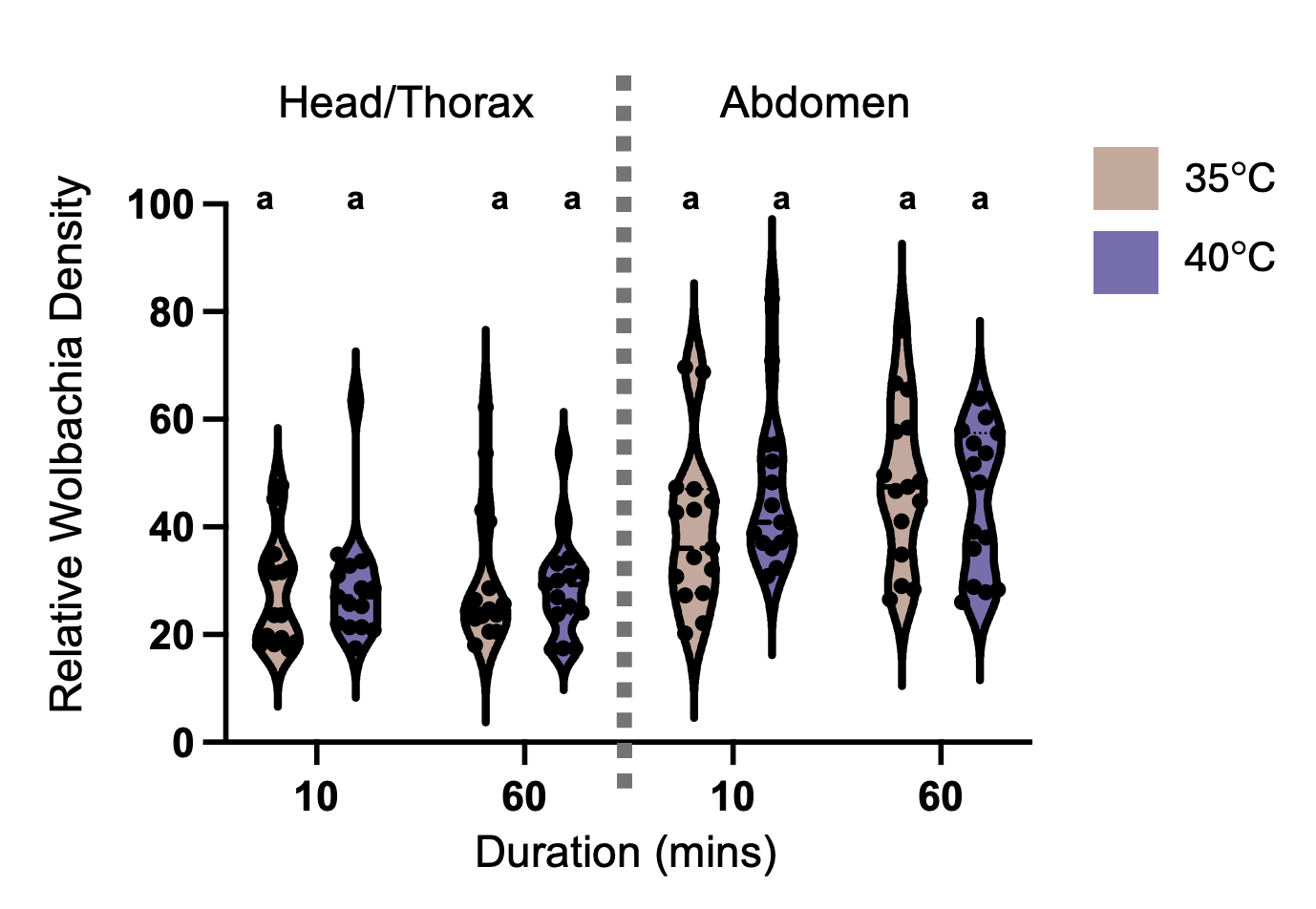


**Figure S2.** Effect of repeated thermal exposure on *Wolbachia* density in DENV-2 infected mosquitoes. The effect of heat shock events with varying durations (10 or 60 minutes) and intensities (35°C or 40°C) on *Wolbachia* density relative to the housekeeping gene *RpS17*, with 15 individuals per treatment. These mosquitoes have been exposed to three heat shock events. Violin plots display the distribution of knockdown times, with the thick black bar representing the median and the dotted lines indicating the interquartile range. Dots represent the individual head/thorax or abdomen. Different letters (a,b,c) indicate statistically significant differences (p<0.05) among treatment groups as determined by Tukey’s post hoc test.


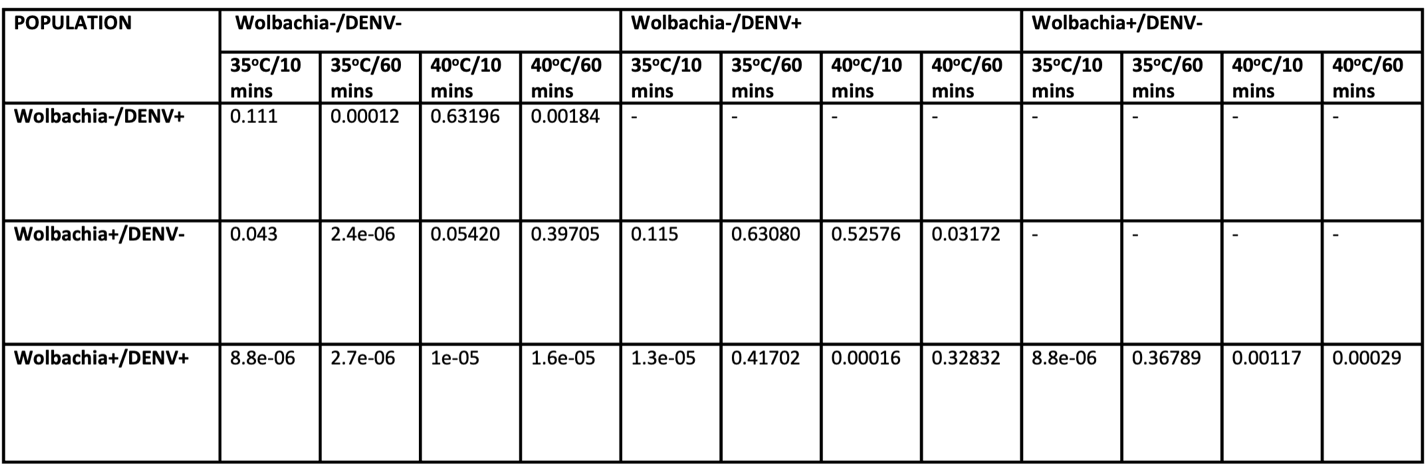


**Table S1.** Log-rank p-values from pairwise comparison analysis of survival in four *Ae.aegypti* populations exposed to three repeated thermal exposure events.


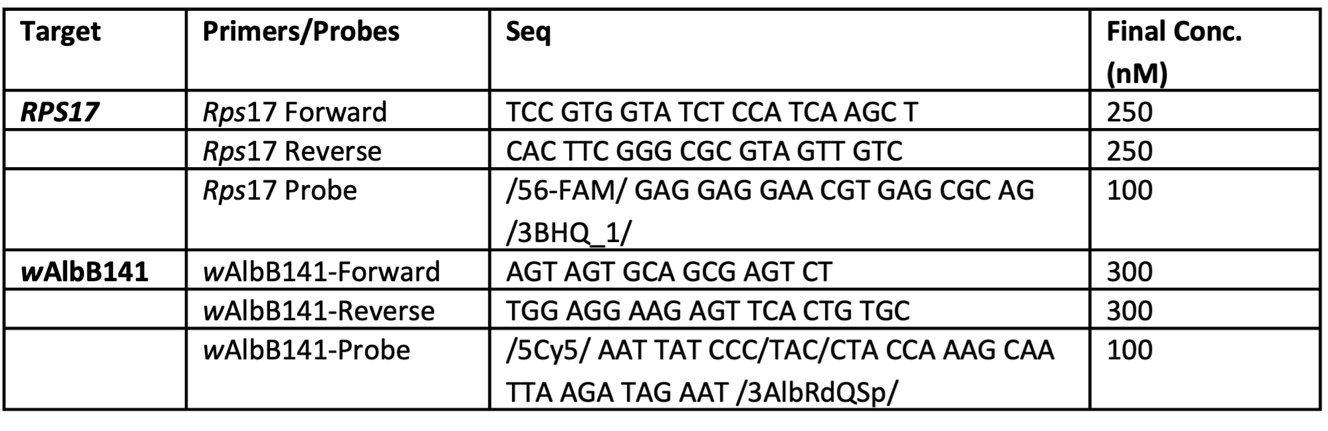


**Table S2.** Sequences and final concentrations of primers and probes used for qPCR quantification of *Wolbachia* and the mosquito housekeeping gene (*RpS17*).
